# β‐Cell gene expression stress signatures in types 1 and 2 diabetes

**DOI:** 10.1111/1753-0407.70026

**Published:** 2024-11-06

**Authors:** Xiaoyan Yi, Decio L. Eizirik

**Affiliations:** ^1^ ULB Center for Diabetes Research, Medical Faculty Université Libre de Bruxelles Brussels Belgium

**Keywords:** apoptosis, endoplasmic reticulum stress, gene signatures, inflammation, senescence, type 1 diabetes, type 2 diabetes, β‐cells

Diabetes mellitus (DM) is a chronic metabolic disorder that occurs when pancreatic β‐cells can no longer produce enough insulin to maintain normal blood glucose levels. DM presently affects 10.5% of the world adult population. While T1D is a disease of “mistaken identity,” where the immune system attacks and destroys pancreatic β‐cells in the context of islet inflammation (insulitis),^1^ T2D is associated with sedentary lifestyles and high‐fat diets, typically involving ineffective use of insulin and progressive loss of β‐cell function.^1^ Both diseases result from multifaceted interactions between genetic and environmental factors, with β‐cell failure as the core mechanism of pathogenesis.

In T1D, the disease arises from a complex interaction between immune cells and β‐cells, involving chemokine and cytokine release and signals from stressed or dying β‐cells that attract and activate immune cells to the islets and lead to β‐cell apoptosis.^2^ Beyond the destruction of β‐cells by the immune system, it is now accepted that stress and impaired function of these cells significantly contribute to the onset and progression of the disease.^1^, ^2^, ^3^ In T2D, the disease is driven by an interplay between insulin resistance and β‐cell dysfunction in genetically susceptible individuals, with metabolic stress and perhaps also inflammation impairing insulin secretion and eventually β‐cell survival, although to a less degree than in T1D.^1^, ^4^, ^5^

The complexity of diabetes pathogenesis makes it very difficult to identify specific causes of the disease, which hampers the development of adequate therapies to protect β‐cells and thus prevent disease. This difficulty was well described by Tolstoy, in his masterpiece “War and Peace,” published 1869 (in this case addressing the Napoleonic war against tsarist Russia): “…the impulse to seek causes is innate in the soul of man. And the human intellect, with no inkling on the immense variety and complexity of circumstances conditioning a phenomena, any one of which may be separately conceived of as the cause of it, snatches the first and most easily understood approximation, and says here is the cause.” In the context of pathophysiology, this had led to the simplistic view of “one gene, one protein, one disease.” However, with the sequencing of the human genome and the subsequent advent of omics technologies that allow interrogating the whole system in a parallel and often also in a sequential way, our understanding of complex diseases changed: we now focus on the dysfunction of gene and transcription factor networks and of post‐transcriptional and post‐translational mechanisms.

The advent of single‐cell RNA sequencing (scRNA‐seq) has provided a new tool for dissecting the molecular intricacies underlying pancreatic islet cells stress and thus addressing mechanisms of disease closer to its real “immense variety and complexity of circumstances.” A recent study by Maestas et al. focused on β‐cell stress by utilizing in vitro models to investigate the effects of ER stress inducers (thapsigargin, brefeldin A) and inflammatory cytokines (IFNγ, IL1β, TNFα, and their combination) on β‐cells, using islets from five donors for the scRNA‐seq analysis.^6^ This study provided very interesting information, but the limited number of donors and the use of in vitro stress conditions to model T1D and T2D may have not fully captured the in vivo disease context.

To further investigate the stress signatures potentially present in the human disease, we presently analyzed data from the Human Pancreas Analysis Program (HPAP).^7^, ^8^ The HPAP provides an extensive public database with scRNA‐seq data from islets from non‐diabetic individuals or individuals affected by T1D or T2D, offering a valuable resource to study disease‐specific transcriptional profiles of β‐cells.

We thus re‐analyzed scRNA‐seq data from the HPAP database up to 12.2023, which includes 10X Genomics data for islets from 27 non‐diabetic, 7 T1D, and 10 T2D individuals, using our previously described pipeline.^9^ We employed an indexed gene signature scoring method,^9^, ^10^, ^11^ to profile six sets of β‐cell stress signatures, namely, inflammation, senescence, autophagy, apoptosis, endoplasmic reticulum (ER) protein processing, and unfolded protein response (UPR). The gene signature for inflammation was collected from our previous study, which comprises 80 genes highly stimulated (i.e., >3 fold) by IFN‐α, IFN‐γ, and IL‐1β in human insulin‐producing EndoC‐βH1 cells.^9^ The remaining five sets of gene signatures are derived from the Reactome and Kyoto Encyclopedia of Genes and Genomes (KEGG) databases and included the following number of genes: 157 for cellular senescence; 146 for autophagy; 140 for apoptosis; 170 for ER protein processing; 92 for UPR.

A potential limitation for the present analysis is the diverse number of cells recovered from the three groups (15 281 for 27 non‐diabetic controls, 585 for 7 T1D, and 1455 for 10 T2D), which is due to both the different number of donors and the inherent loss of β‐cells in the course of diabetes (associated to the difficult of isolating islets from individuals affected by diabetes). In spite of this methodological limitation, our analysis revealed that all β‐cell stress signatures were upregulated in both T1D and T2D, with T1D showing higher scores for all forms of stress (Figure 1). Notably, T1D β‐cells exhibited a >200% increase in the inflammation signature score as compared to T2D or non‐diabetic controls. There was also a clear increase in the score signatures of senescence, autophagy, apoptosis and ER stress in β‐cells from individuals affected by T1D compared to non‐diabetic individuals (20%–43%), and only a mild (6%–27%) increase in β‐cells from individuals affected by T2D compared to controls. These results confirm and extend the observations by Maestas et al.^6^ that in both T1D and T2D β‐cells experience multiple forms of stress, while emphasizing that β‐cells in T1D undergo a more severe stress, which is in line with the faster and more massive β‐cell loss in T1D as compared to T2D.^5^

**FIGURE 1:**
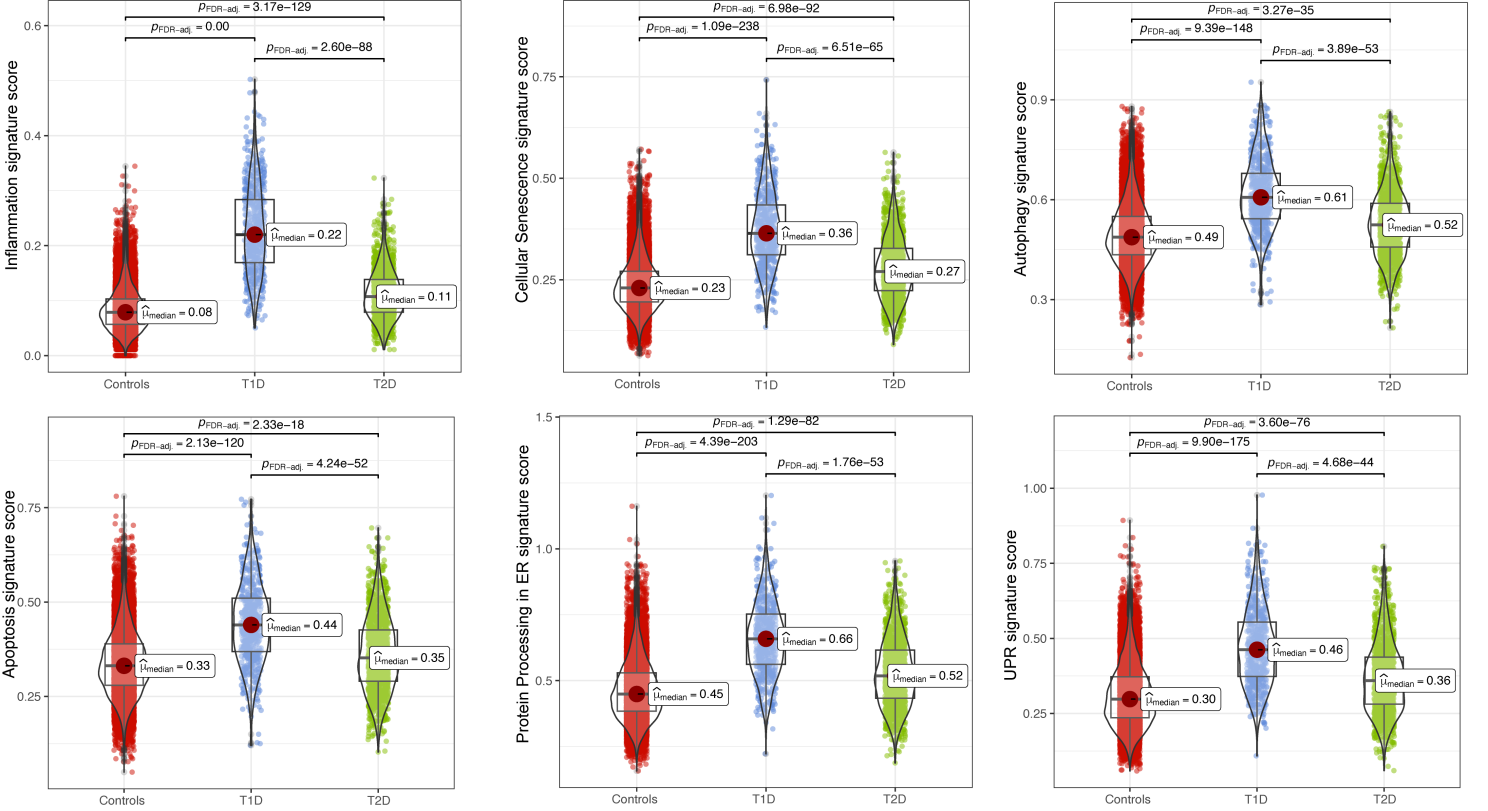
Elevated β‐cell stress gene signatures in individuals with T1D and T2D compared to healthy controls. The score was calculated as the average expression of genes within each gene signature, which was obtained from Reactome, KEGG, and our previous publication.^9^ A Kruskal‐Wallis test was performed, followed by a two‐tailed Dunn test with *p* values adjusted using the false discovery rate (FDR). The number of β‐cells retrieved from HPAP single‐cell datasets (https://hpap.pmacs.upenn.edu/) was as follows: 15 281 for 27 non‐diabetic controls, 585 for 7 T1D, and 1455 for 10 T2D. ER, endoplasmic reticulum; UPR, unfolded protein response.

Proper insulin processing in the ER under metabolic stress conditions necessitates physiological and transient activation of the UPR, while prolonged and excessive activation (“terminal” UPR) can trigger cell death.^5^ To understand the relationship between the UPR and apoptosis or cellular senescence in diabetes, we conducted a correlation analysis using the above described signature index scores. There was a significant positive correlation between the UPR and both apoptosis and cellular senescence in T1D and T2D (Figure 2), with the strongest correlation observed in T1D (Figure 2A), which is in line with the detection by histology of ER stress markers in islets of individuals affected by T1D.^12^ To further understand the causality of apoptosis, we developed a multiple regression model with the following formulations: apoptosis ~ inflammation + autophagy + cellular senescence + UPR. We found that these stress signaling pathways together effectively predict cell death signature in T1D (*R* ^2^ = 0.80) and T2D (*R* ^2^ = 0.75).

**FIGURE 2:**
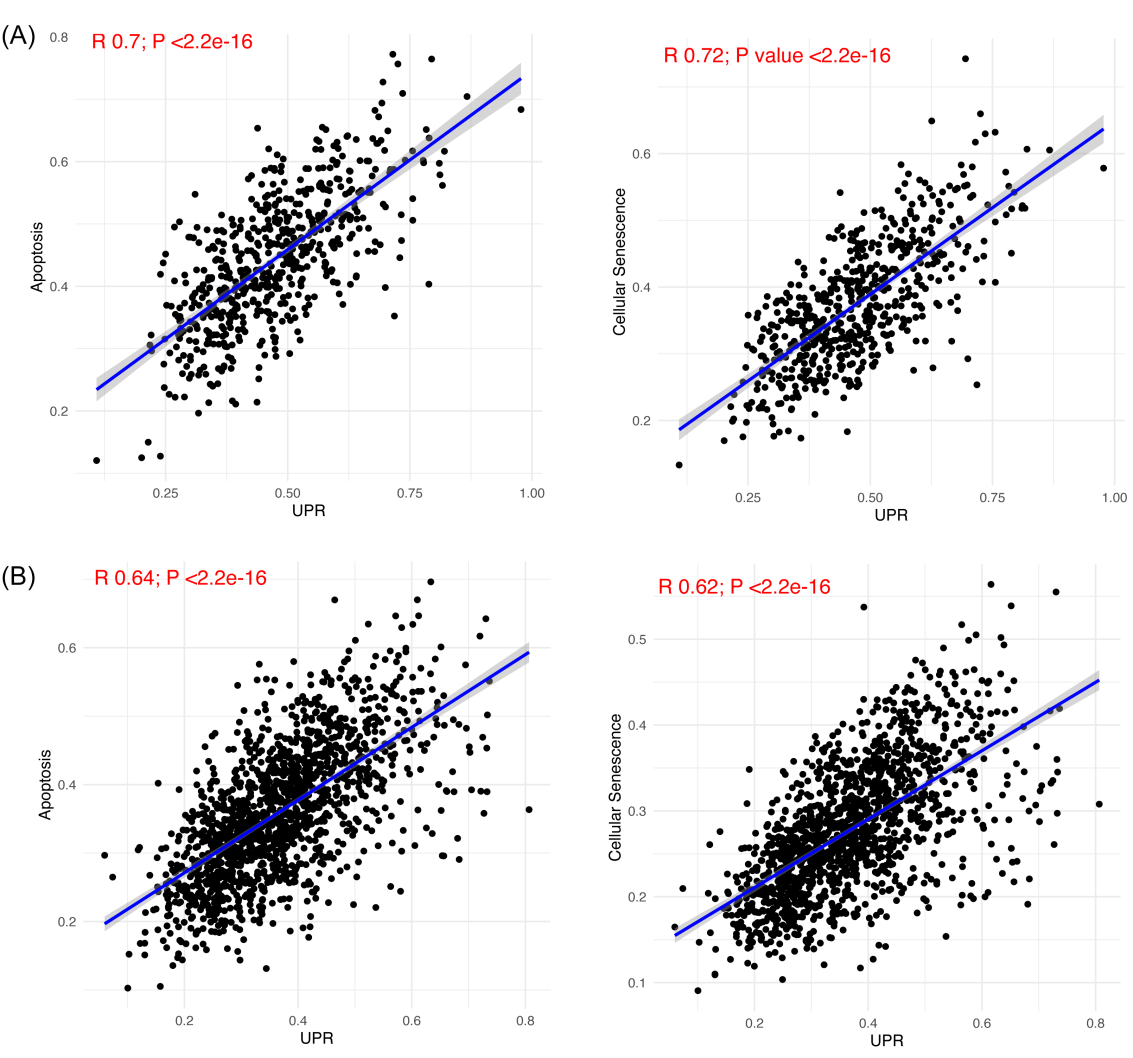
The correlation between β‐cell UPR signaling and apoptosis and cellular senescence signatures in T1D and T2D. Correlation and regression analyses were performed between the UPR gene signature score and both the apoptosis and cellular senescence scores using Pearson correlation. The correlation coefficients, statistical significance, and 95% confidence intervals are displayed for each plot.

The implications of ours and Maestas et al.^6^ findings are twofold. First, targeting β‐cell stress pathways—particularly inflammation, ER stress, and senescence—may offer a therapeutic strategy for T1D and, to a less extent, to T2D. These observations, however, must be considered with caution as for instance gene signatures alone are not sufficient to identify senescence and many markers of the secretory phenotype of senescence, downstream of the transcription factors NF‐κB and STATs,^13^ are also part of the autoimmune‐induced insulitis,^2^, ^5^ making it difficult to discriminate between senescence‐ and inflammation‐induced signatures in T1D.

In support for a role of components of senescence contributing to T1D is the demonstration that targeted elimination of senescent β‐cells prevent diabetes in non‐obese (NOD) diabetic mice,^14^ and the fact that an early senescence signature is present in the residual β‐cells of patients with T1D.^15^

The present analysis also indicated a positive correlation between the UPR and apoptosis, as well as between the UPR and cellular senescence in T1D and, to a less extent, T2D. Excessive and/or prolonged UPR contributes to the development of diabetes by promoting pancreatic β‐cell loss and insulin resistance.^16^ IRE1, one of the UPR's master regulators, induces β‐cell apoptosis and degeneration at “terminal” ER stress level, while inhibition of IRE1 in mouse models protects β‐cells and may provide therapeutic opportunities for diabetes.^17^ Moreover, inhibition of another UPR regulator, namely the eIF2α kinase PERK, reverses the translation blockade present in stressed human islets and prevents diabetes in NOD mice.^18^

Of interest, there is also crosstalk between the different β‐cell stresses, as deletion of the UPR genes ATF6 and IRE1α in NOD mice before the onset of insulitis leads to a p21‐driven early senescence phenotype that paradoxically reduces terminal β‐cell senescence and the incidence of diabetes.^15^ Future research should explore the interactions between the stress signaling pathways and their leading‐edge genes discussed above, as well as their combined impact on β‐cell function and survival across different types of diabetes.

This comment highlights the β‐cell stress signatures in T1D and T2D, utilizing an index score method based on scRNA‐seq data from 44 human islets donors. Key findings indicate that T1D (and to a less extent T2D) is characterized by elevated inflammatory stress and disturbances in multiple stress signaling pathways. Strong correlations was observed between the UPR, apoptosis, and cellular senescence. These results add relevant human disease information to the previous in vitro findings by Maestas et al.^6^ and emphasize the relevance of studying gene signatures of affected human tissues in autoimmune or degenerative diseases in the search for better and more targeted therapies that address the disease at its real level of complexity.^10^, ^19^

## AUTHOR CONTRIBUTIONS

Decio L. Eizirik conceptualized the study, Xiaoyan Yi performed data analysis and drafted the manuscript. Decio L. Eizirik contributed by reviewing, editing, and adding content. Both authors approved the final version of the manuscript. Xiaoyan Yi and Decio L. Eizirik have contributed significantly and in keeping with the latest guidelines of International Committee of Medical Journal Editors. Xiaoyan Yi and Decio L. Eizirik serve as guarantors of this work.

## FUNDING INFORMATION

Research by the authors is supported by grants from Breakthrough T1D (formerly JDRF International (3‐SRA‐2022‐1201‐S‐B [1] and 3‐SRA‐2022‐1201‐S‐B [2])); the National Institutes of Health ‐ Human Islet Research Network Consortium on Beta Cell Death & Survival from Pancreatic β‐Cell Gene Networks to Therapy (HIRN‐CBDS) (grant U01 DK127786); and the National Institutes of Health NIDDK grants, RO1DK126444 and RO1DK133881‐01.

## CONFLICT OF INTEREST STATEMENT

The authors declare no conflicts of interest related to this commentary.
